# Colonic stent as a bridge to surgery versus emergency rection for malignant left-sided colorectal obstruction: A systematic review and meta-analysis of randomized controlled trials

**DOI:** 10.1097/MD.0000000000036078

**Published:** 2023-12-15

**Authors:** Rumin Shang, Xiangming Han, Cui Zeng, Fei Lv, Rong Fang, Xiaochang Tian, Xiangwu Ding

**Affiliations:** a Department of Gastroenterology, Wuhan Pu’ai Hospital, Tongji Medical College, Huazhong University of Science and Technology, Wuhan, China; b Department of Gastroenterology, Wuhan Fourth Hospital, Wuhan, China; c Department of Oncology, The First Affiliated Hospital of Soochow University, Suzhou, China.

**Keywords:** emergency rection, endoscopic self-expanding metal stent implantation, left-sided malignant colorectal obstruction, long-term oncological outcomes, surgical bridge

## Abstract

**Introduction:**

The role of self-expanding metal stent (SEMS) implantation as a bridge to surgery in malignant left-sided colorectal obstruction (MLCO) remains controversial.

**Objective:**

To evaluate the safety of SEMS implantation versus emergency surgery (ER) in the treatment of MLCO.

**Methods:**

Four major literature databases (Cochrane Library, Embase, PubMed, and Web of Science) were searched to collect articles published before April 20, 2023. After determining random or fixed-effect models based on heterogeneity tests, odds ratios (RR) or standardized mean differences (SMD) with their respective 95% confidence intervals (CI) were calculated.

**Results:**

Nineteen randomized controlled studies were included. The main outcomes included overall tumor recurrence rate, 30-day mortality rate, and overall incidence of complications. Secondary outcomes included mortality-related indicators, tumor recurrence-related indicators, surgery-related indicators, and other relevant indicators. The study found that there was no significant difference in the 30-day mortality rate between the SEMS group and the er group. However, the SEMS group had a lower overall incidence of complications (RR = 0.787, *P* = .004), lower incision infection rate (RR = 0.472, *P =* .003), shorter operation time (SMD = −0.591, *P* = .000), lower intraoperative blood loss (SMD = −1.046, *P* = .000), lower intraoperative transfusion rate (RR = 0.624, *P =* .021), lower permanent stoma rate (RR = 0.499, *P =* .000), lower overall stoma rate (RR = 0.520,*P* = .000), shorter hospital stay (SMD = −0.643, *P =* .014), and more lymph node dissections during surgery (SMD = 0.222, 95% CI: 0.021–0.423, *P =* .031), as well as a higher primary anastomosis rate (RR = 0.472, 95% CI: 0.286–0.7 77, *P =* .003), among other advantages. However, the SEMS group had a higher overall tumor recurrence rate (RR = 1.339, *P =* .048).

**Conclusion:**

SEMS has significant advantages over er in relieving clinical symptoms and facilitating postoperative recovery in MLCO, but does not reduce the tumor recurrence rate. Neoadjuvant chemotherapy combined with SEMS may provide a new approach to the treatment of MLCO.

## 1. Introduction

Colorectal cancer (CRC) is one of the common malignant tumors in the digestive system.^[1]^ Recently, it has been reported that CRC is the third most common cancer in the world but the second leading cause of death.^[2]^ Malignant colorectal obstruction accounts for 8% to 34% of patients with CRC and is a life-threatening illness that requires immediate surgical intervention.^[3]^ Emergency surgical treatment is often associated with high permanent stoma rates, high mortality rates, and high rates of complications. This may be due to varying degrees of circumferential growth of late-stage tumors that block the intestinal lumen or cause narrowing of the intestinal lumen due to inflammation and swelling around the tumor, leading to continuous accumulation of intestinal contents, sustained increases in intestinal pressure, obvious intestinal wall edema, disturbances in intestinal blood circulation, and secondary electrolyte imbalances, acid-base imbalances, peritonitis, intestinal perforation, and septic shock.^[4]^

20 years ago, the first implantation of self-expanding metallic stent (SEMS) as a “surgical bridge” to restore left colonic malignant obstruction patients was applied in clinical practice.^[5]^ SEMS, as a preoperative transitional means, provides sufficient conditions for the recovery of the patient intestinal function and adjustment of nutritional status. A meta-analysis showed that 78% of patients receiving stent treatment were able to undergo single-stage surgery with anastomosis, significantly improving the quality of life of patients.^[6]^ Although SEMS is now widely used, clinical trials have produced conflicting results, mainly because it may be detrimental to the long-term tumor outcome of curable diseases.^[7]^ The 2017 World Emergency Surgery Society Guidelines recognize the advantages provided by SEMS but emphasize that its use in patients who are treatable by surgery may expose some long-term tumor problems.^[8]^ In contrast, the recent European Society for Gynaecological Endoscopy guidelines recommend the use of SEMS because it is associated with lower mortality rates, shorter hospital stays, and lower colostomy rates.^[9]^ Currently, there are few meta-analyses based on randomized controlled trials (RCTs) to evaluate the safety and effectiveness of SEMS in treating MLCO, which prevents the drawing of clear conclusions. The last systematic review, which only included 12 RCT studies, was published 2 years ago.^[10]^ Therefore, we conducted an RCT-based meta-analysis to evaluate whether SEMS is a safe alternative in er for MLCO.

## 2. Materials and methods

### 2.1. Retrieval strategy

We searched 4 major literature databases (Cochrane Library, Embase, PubMed, and Web of Science) for English articles published since April 20, 2023. The search terms included “Colorectal cancer,” “self-expandable metallic stents,” “intestine obstruction,” and “surgery,” and other terms such as “Colorectal Neoplasm,” “Self-expanding metal bracket,” “colonic obstruction,” and “emergency surgery” were added as free terms. To improve the search results, we combined the subject terms with the free terms. To avoid omissions, we also manually searched the reference lists of relevant literature in online databases and previously published systematic reviews to further identify relevant studies.

### 2.2. Inclusion and exclusion criteria

Inclusion criteria: study subjects: patients with acute obstruction of the left side of the colon-rectum due to CRC who require emergency decompression; intervention measures: endoscopic stent implantation or emergency surgery to relieve intestinal obstruction; reporting outcome indicators of interest to us; study type: randomized controlled clinical trials. Exclusion criteria: review articles; animal experiments; conference papers; articles from which corresponding data cannot be extracted through various ways.

### 2.3. Data extraction and literature quality assessment

We imported the retrieved literature into Endnote X9.0 software and 2 authors independently screened and removed duplicates based on the titles and abstracts of the literature. For articles with different opinions, a third-party researcher made the final judgment after discussion. The entire operation process followed the PRISMA statement guidelines and standardized forms were used for data extraction, including the name of the primary author, publication year, study region, research quality score, study design type, surgical purpose (curative or palliative), single/multi-center, stent dwell time, sample size, gender, age, outcome indicators, etc. In evaluating the included randomized controlled trials, we used the RCT bias evaluation tool recommended by the Cochrane Collaboration Network, which includes 7 items: randomization method; allocation concealment; blinding of subjects; blinding of outcome assessment; completeness of outcome data; selective reporting of study results; other sources of bias. For each item, we made low risk, unclear risk, and high-risk evaluations separately.

### 2.4. Statistical analysis

For continuous data, if the included literature used the median (quartile) to express, we used the optimal sample mean estimation method proposed by Luo et al to convert to the mean,^[11]^ and used the sample standard deviation estimation method proposed by Wan et al to calculate the standard deviation.^[12]^ We used the standardized mean difference (SMD) for statistical analysis of metric data, and for binary data, we used the Mantel-Haenszel method to calculate the merged relative risk (RR) and respective 95% confidence interval (CI). Cochran Q test and *I*^2^ value were used to determine heterogeneity, and if there was no statistical heterogeneity among the included studies (*P* ≥ .05, *I*^2^ ≤ 50%), fixed-effect models were used for meta-analysis; if there was significant heterogeneity among the studies, random-effect models were used. We also conducted subgroup analysis and sensitivity analysis for the main effect to identify the source of heterogeneity. Funnel plot and Begger test were used to qualitatively and quantitatively analyze publication bias. An asymmetric funnel plot or *P* < .05 suggested publication bias.

## 3. Result

### 3.1. Included literature characteristics

**Figure 1.**
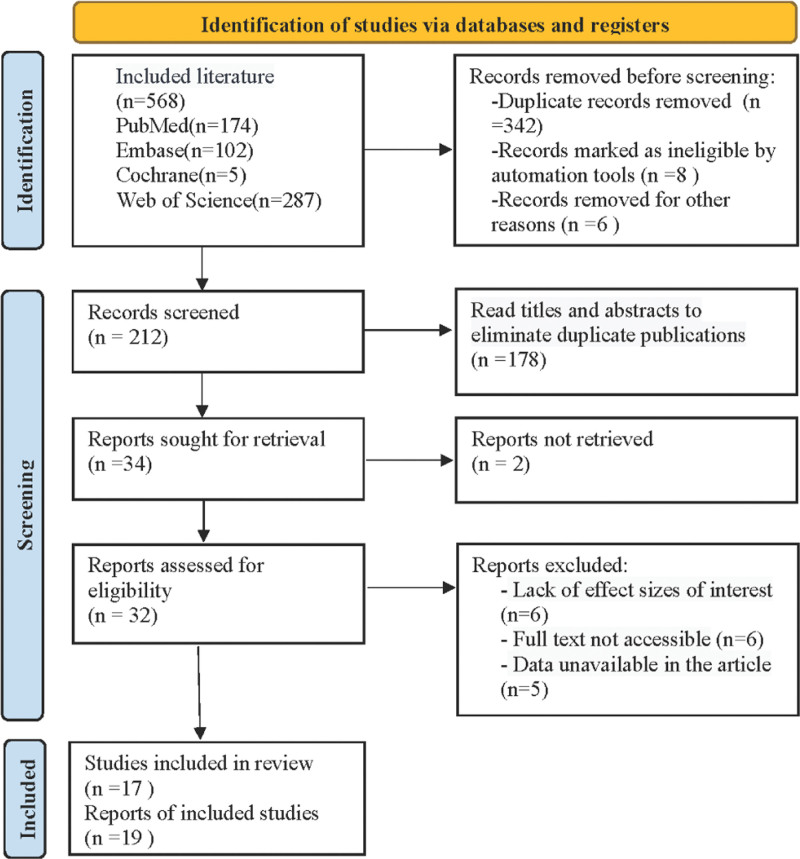
The research conducted by Hill and Cui respectively included 2 randomized controlled studies.

**Figure 2.**
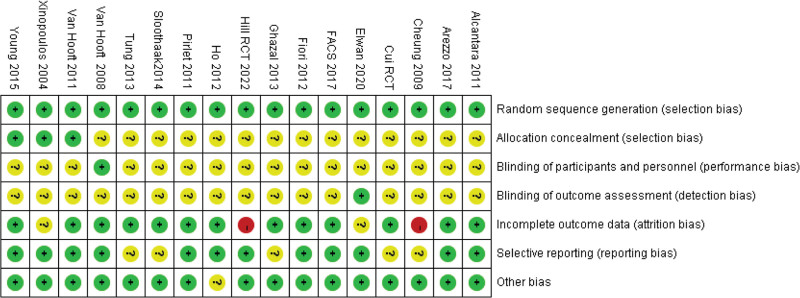
Bias Risk Assessment Graph.

Based on a preliminary search, 568 relevant studies were identified. First, 356 studies were removed through Endnote based on title duplication. The remaining 212 studies were screened based on the titles and abstracts. Additionally, 180 studies based on animal experiments, review articles, and case reports were excluded from this study. A comprehensive review was performed on the remaining 32 studies. Six studies were excluded due to lack of the interested effect size, 6 studies were excluded due to unavailability of the full text, and 5 studies had unavailable data. Finally, 17 articles were included in this meta-analysis.^[13–29]^ Hill and Cui studies were each included in 2 randomized controlled studies, thus a total of 20 studies were included in the meta-analysis. The process is shown in Figure 1, and the basic characteristics of the included studies are shown in Table 1. Table 2 summarizes the basic characteristics of the included studies. A total of 19 studies were included, published between 2004 and 2022, and involving 1094 participants. These studies were conducted in Asia (4 in China, 1 in Singapore), Africa (2 in Egypt), Europe (3 in the Netherlands, 2 in Italy, 2 in the UK, 1 in France, 1 in Spain, 1 in Greece), and Oceania (2 in Australia). The risk of bias assessment items for each included study are shown in Figure 2.

**Table 1:** Table of basic literature characteristics.

Author	Yr	Country	Area	Study type	Intervention type	Single/multi-center	Age (yr)		Gender (male/female, number)	
							SEMS	ER	SEMS	ER
Young	2015	Australia	Oceania	RCT	Palliative	multi	41–83	35–86	9/17	8/18
Sloothaak	2014	Netherlands	Europe	RCT	curative	multi	60–67	61–79	12/14	18/14
Alcantara	2011	Spain	Europe	RCT	curative	single	63–81	62–80	5/10	7/6
Arezzo	2017	Italy	Europe	RCT	curative	multi	43–90	44–94	28/28	32/27
Ho	2012	Singapore	Asia	RCT	curative	single	51–85	49–84	7/13	9/10
Pirlet	2011	France	Europe	RCT	curative	multi	60–81	63–86	16/14	13/17
Van Hooft	2011	Netherlands	Europe	RCT	curative	multi	58–82	62–81	24/23	27/24
Van Hooft	2008	Netherlands	Europe	RCT	Palliative	multi	46–81	42–88	4/7	7/3
Cheung	2009	China	Asia	RCT	curative	single	39–68	27–86	14/10	12/12
Ghazal	2013	Egypt	Africa	RCT	curative	single	37–68	35–66	18/12	11/19
Xinopoulos	2004	Greece	Europe	RCT	curative	single	64–87	64–87	9/6	7/8
Fiori	2012	Italy	Europe	RCT	Palliative	single	77.2	76	6/5	7/4
Hill RCT1	2022	The UK	Europe	RCT	curative	multi	61–89	62–78	34/64	40/68
Hill RCT2	2022	The UK	Europe	RCT	Palliative	multi	61–89	62–78	6/8	5/9
FACS	2017	Sydney	Oceania	RCT	Palliative	multi	53–79	53–79	NA	NA
Cui RCT1	2011	China	Asia	RCT	curative	single	49–75	29–79	8/7	9/11
Cui RCT2	2011	China	Asia	RCT	curative	single	49–77	29–79	8/6	9/11
Tung	2013	China	Asia	RCT	curative	single	27–36	39–68	14/10	12/12
Elwan	2020	Egypt	Africa	RCT	curative	multi	33–72	35–68	20/10	12/18

RCT = randomized controlled trial, SEMS = self-expanding metal stent.

**Table 2:** **S**ubgroup analysis of main effect size.

Group	Number of study cases	RR (95% CI)	*P*	Heterogeneity test	
				*P*	*I^2^*(%)
Subgroup analysis of overall tumor recurrence rate					
Total number	7	1.339 (1.002, 1.788)	.048	.096	44.3
Intervention measures					
Palliative	1	0.886 (0.481, 1.634)	.699	NA	NA
Curative	6	1.339 (1.002, 1.788)	.039	.106	45.0
Single/multi-center					
Single	3	2.591 (1.298, 5.173)	.007	.408	0
Multi	4	1.103 (0.799, 1.521)	.552	.276	22.5
Subgroup analysis of 30-d mortality rate					
Total number	6	0.978 (0.548, 1.746)	.941	.425	0
Intervention measures					
Palliative	1	0.500 (0.100, 2.496)	.840	NA	NA
Curative	5	1.084 (0.579, 2.029)	.250	.378	5
Single/multi-center					
Single	2	1.484 (0.423, 5.206)	.537	.075	68.5
Multi	4	0.857 (0.444, 1.652)	.645	.747	0
Area					
Oceania	1	0.500 (0.100, 2.496)	.232	NA	NA
Europe	4	1.262 (0.653, 2.441)	.489	.420	0
Asia	1	0.190 (0.011, 3.416)	.260	NA	NA
Subgroup analysis of overall complication rate					
Total number	10	0.787 (0.667, 0.928)	.004	.038	49.4
Intervention measures					
Palliative	2	0.248 (0.391, 1.305)	.138	.393	0
Curative	8	0.802 (0.676, 0.953)	.012	.021	57.6
Single/multi-center					
Single	5	0.362 (0.229, 0.572)	0	.599	0
Multi	5	0.944 (0.790, 1.127)	.521	.721	0
Area					
Oceania	1	0.714 (0.391, 1.305)	.271	NA	NA
Europe	6	0.913 (0.759, 1.097)	.331	.305	16.8
Asia	2	0.472 (0.270, 0.825)	.008	.336	0
Africa	1	0.276 (0.104, 0.733)	.010	NA	NA

### 3.2. Meta-analysis results

#### 3.2.1. Main effect size.

In Fig. 4, RRs from 19 studies were extracted after multivariable correction. Seven studies^[13–15,19,22,23]^ including 529 patients reported the overall tumor recurrence rate of 2 groups of patients. After heterogeneity test (*I*^2^ = 44.3%, *P =* .096) which indicated no significant heterogeneity among the selected studies, a fixed-effect model was used to pool the effect size. The summary results showed that the overall tumor recurrence rate in the stent group was higher than that in the emergency surgery group (RR = 1.339, 95% CI: 1.002–1.788; *P =* .048). Six studies involving 543 patients reported 30-day mortality rates for patients in the stent and emergency surgery groups.^[13–15,20,24,27]^ After heterogeneity test (*I*^2^ = 0.00%, *P =* .425), which indicated small heterogeneity between the selected studies, a fixed-effect model was used to pool the effect size. The summary results showed that the difference in 30-day mortality rates between the 2 groups was not significant (RR = 0.978, 95% CI: 0.548–1.746; *P =* .941). Ten studies reported a total complication rate among 735 patients.^[13–15,17,19–21,24,26–28]^ After heterogeneity test (*I*^2^ = 49.4%, *P =* .038), which indicated no significant heterogeneity among the selected studies, a fixed-effect model was used to pool the effect size. The summary results showed that the overall complication rate in the stent group was lower than that in the emergency surgery group (RR = 0.787, 95% CI: 0.667–0.928; *P =* .004).

##### 3.2.1.1. Subgroup analysis.

We subsequently conducted subgroup analyses of the main effect size, with stratification factors including intervention measures, single or multi-center studies, and geographic regions. For overall tumor recurrence rate, in the subgroup analysis stratified by intervention measures, only 1 article reported no statistically significant difference between SEMS and ER groups for patients receiving palliative treatment. In patients receiving curative treatment, the overall tumor recurrence rate was higher in the stent group than in the surgery group, and the difference was statistically significant. In the subgroup analysis stratified by single or multi-center studies, data from single-center studies showed a higher overall tumor recurrence rate in the SEMS group than in the ER group, while data from multi-center studies showed no statistically significant difference, indicating that single or multi-center studies may be associated with overall tumor recurrence rate. For 30-day mortality rate, no significant heterogeneity was found in the 3 subgroup analyses, indicating that they were not the reasons for heterogeneity in 30-day mortality rate. For overall complication rate, in patients receiving curative treatment, the overall complication rate in the SEMS group was lower than that in the ER group, and the difference was statistically significant. Conversely, in patients receiving palliative treatment, there was no statistically significant difference in the incidence of complications between the 2 groups. In the subgroup analysis stratified by single or multi-center studies, the result was the same as that for overall tumor recurrence rate. In the subgroup analysis stratified by geographic regions, data from Europe and Oceania showed no statistically significant difference between the SEMS and ER groups in terms of overall complication rate, while data from Asia and Africa showed that the overall complication rate in the SEMS group was lower than that in the ER group, indicating that all 3 stratification factors were associated with the overall complication rate. The specific results are shown in Table 2.

##### 3.2.1.2. Sensitivity analysis.

The results of the sensitivity analyses of the main effect size were shown in Figure 5, with a focus on the results of 30-day mortality rate and overall complication rate. The results showed that most of the pooled results from the remaining studies did not change after individual studies were removed. Only for the overall tumor recurrence rate, the difference between the 2 groups was not statistically significant (RR = 1.210, 95% CI: 0.895–1.636, *P =* .216) after the study by Tung et al^[23]^ was removed, which suggested that the stability of the results of this study was slightly lower.

**Figure 3.**
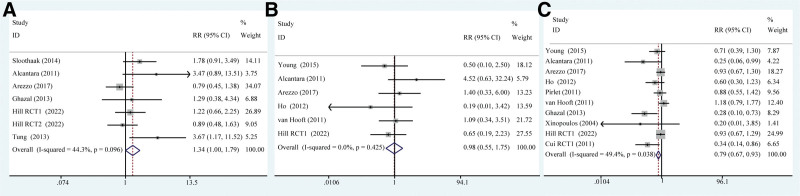
Main effect size forest plot. (A) Comparison of overall tumor recurrence rates between 2 groups. (B) Comparison of 30-d mortality rates between 2 types. (C) Comparison of overall complication rates between 2 groups.

**Figure 4.**
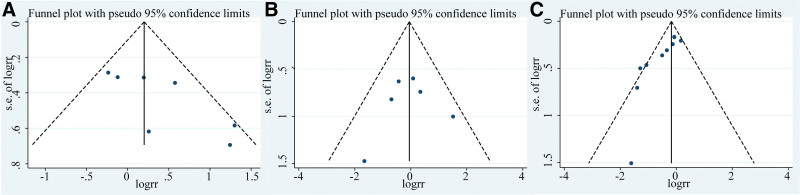
Main effect size funnel plot. (A) Comparison of overall tumor recurrence rates between 2 groups. (B) Comparison of 30-d mortality rates between 2 groups. (C) Comparison of overall complication rates between 2 groups.

**Figure 5.**
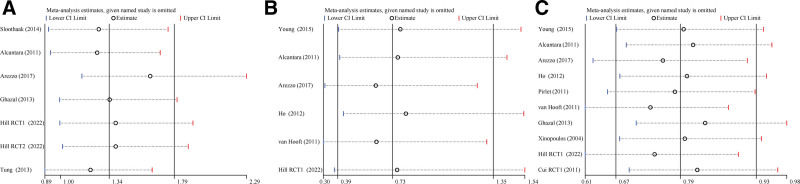
Sensitivity analysis of main effect size. (A) Comparison of overall tumor recurrence rates between 2 groups. (B) Comparison of 30-d mortality rates between 2 types. (C) Comparison of overall complication rates between 2 groups.

##### 3.2.1.3. Publication bias.

We conducted a funnel plot analysis of the main effect size, as shown in Figure 3. The points in the funnel plot were generally within the funnel, indicating an acceptable level of publication bias. To quantify publication bias more accurately, we conducted Begger test for the main effect size. The results showed no significant publication bias in 30-day mortality rate between the 2 groups (*P* = .453), but there was publication bias in the overall complication rate (*P* = .002) and the overall tumor recurrence rate (*P* = .035). This may be due to negative results not being published, or may be related to inconsistent follow-up times in the stent and surgery groups, a large proportion of loss to follow-up due to long follow-up times in some RCTs, and small sample sizes in some trials. This suggests that more large-sample, high-quality RCTs are needed to confirm these results.

#### 3.2.2. Other effect size.

##### 3.2.2.1. Mortality-related indicators.

Three studies including 384 patients reported the 1-year mortality rate.^[13,15,27]^ The combined data showed no statistically significant difference between the ER group and the SEMS group (RR = 1.103, 95% CI: 0.804–1.515, *P =* .543). Three studies including 390 patients reported the 3-year mortality rate.^[13,15,22]^ The combined data showed no statistically significant difference between the ER group and the SEMS group (RR = 1.003, 95% CI: 0.866–1.162, *P =* .964). See Table 3 for specific details.

**Table 3:** Indicators related to mortality rate.

Effect size	Studys	numbers	Heterogeneity test	Analysis model	RR/SMD	95%CI	*P*
1-yr mortality rate	3	384	*P* = .436	Fixed-effects model	1.103	(0.804, 1.515)	.543
			*I*^2^ = 0.0%				
3-yr mortality rate	3	390	*P* = .432,	Fixed-effects model	1.003	(0.866, 1.162)	.964
			*I*^2^ = 0.0%				

SMD = standardized mean differences.

##### 3.2.2.2. Postoperative complication-related indicators.

Our summary results showed that there were no statistically significant differences in the incidence of postoperative intra-abdominal infection (RR = 0.604, 95%CI: 0.166~2.197, *P =* .444), urinary tract infection (RR = 0.568, 95%CI: 0.252~1.285, *P =* .175), heart failure (RR = 0.68, 95%CI: 0.182~2.544, *P =* .567), pulmonary infection (RR = 0.638, 95%CI: 0.313~1.301, *P =* .216), anastomotic fistula (RR = 0.974, 95%CI: 0.507~1.871, *P =* .937), and postoperative intestinal obstruction (RR = 0.678, 95%CI: 0.291~1.580, *P =* .368) between the 2 groups. However, for the incidence of postoperative wound infection, a total of 8 studies reported the incidence of postoperative wound infection in 497 patients,^[14–16,19,20,24,27,29]^ and the combined data showed that the incidence of wound infection was lower in the stent group than in the emergency group, with statistically significant differences (RR = 0.472, 95%CI: 0.286~0.777, *P =* .003). Please see Table 4 for details.

**Table 4:** Indicators related to postoperative complications.

Effect size	Studys	numbers	Heterogeneity test	Analysis model	RR/SMD	95%CI	*P*
Abdominal abscess	5	237	*P* = .857,	Fixed-effects model	0.604	(0.166, 2.197)	.444
			*I*^2^ = 0.0%				
Urinary tract infection	6	422	*P* = .765,	Fixed-effects model	0.568	(0.252, 1.285)	.175
			*I*^2^ = 0.0%				
Heart failure	3	271	*P* = .889,	Fixed-effects model	0.680	(0.182, 2.544)	.567
			*I*^2^ = 0.0%				
Chest infection	7	477	*P* = .783,	Fixed-effects model	0.638	(0.313, 1.301)	.216
			*I*^2^ = 0.0%				
Surgical site infection	8	497	*P* = .802,	Fixed-effects model	0.472	(0.286, 0.777)	.003
			*I*^2^ = 0.0%				
Anastomotic fistula	9	643	*P* = .312,	Fixed-effects model	0.974	(0.507, 1.871)	.937
			*I*^2^ = 15.1%				
Postoperative incidence of intestinal obstruction	7	450	*P* = .845,	Fixed-effects model	0.678	(0.291, 1.580)	.368
			*I*^2^ = 0.0%				

SMD = standardized mean differences.

##### 3.2.2.3. The indicators related to tumor occurrence.

Although our summary results showed that the overall tumor recurrence rate in the stent group was higher than that in the emergency surgery group, 4 studies including 340 patients^[15,19,22,30]^ indicated that the local tumor recurrence rate (RR = 1.08, 95%CI: 0.573~2.034, *P =* .813) did not differ significantly between the stent group and the surgery group. Similarly, for the incidence of liver metastasis reported in 5 studies^[13–15,19]^ (RR = 0.872, 95%CI: 0.505~1.505, *P =* .623), and the incidence of lung metastasis reported in 2 studies^[14,15]^ (RR = 1.91, 95%CI: 0.495~7.368, *P =* .348), there were no statistically significant differences between the stent group and the emergency surgery group. Please refer to Table 5 for details.

**Table 5:** Indicators related to tumor recurrence.

Effect size	Studys	numbers	Heterogeneity test	Analysis model	RR	95%CI	*P*
Local recurrence	4	340	*P* = .404,	Fixed-effects model	1.080	(0.573, 2.034)	.813
			*I*^2^ = 0.0%				
HePatic metastasis	5	447	*P* = .349,	Fixed-effects model	0.872	(0.505, 1.505)	.623
			*I*^2^ = 10.0%				
Pulmonary metastasis	2	143	*P* = .407,	Fixed-effects model	1.910	(0.495, 7.368)	.348
			*I*^2^ = 0.0%				

##### 3.2.2.4. Surgical-related indicators.

Our summary results showed that among 454 patients from 9 studies,^[13,15,16,18–20,27,28]^ the surgical time in the stent group was lower than that in the emergency surgery group, with statistically significant differences (SMD = −0.591, 95%CI: −0.789 to −0.392, *P =* .00). Among 141 patients from 3 studies,^[16,17,19]^ the cumulative intraoperative blood loss in the stent group was lower than that in the emergency surgery group, with statistically significant differences (SMD = −1.046, 95%CI: −1.426 to −0.666, *P =* .00). Among 194 patients from 3 studies,^[15,18,19]^ the transfusion rate in the stent group was lower than that in the emergency surgery group, with statistically significant differences (RR = 0.624, 95%CI: 0.419~0.930, *P =* .003). Among 653 patients from 8 studies,^[13,15,16,20,23,24,29]^ the permanent ostomy rate in the stent group was lower than that in the emergency surgery group, all with statistically significant differences (RR = 0.625, 95%CI: 0.499~0.782, *P =* .00), while among 842 patients from 12 studies,^[13–17,20,21,23,24,27,29]^ the overall ostomy rate in the stent group was lower than that in the emergency surgery group, all with statistically significant differences (RR = 0.52, 95%CI: 0.439~0.614, *P =* .00). Among 412 patients from 5 studies,^[14–16,22,23]^ there were no statistically significant differences in the number of lymph nodes dissected between the stent group and the surgery group (SMD = 0.222, 95%CI: 0.021~0.423, *P =* .031). Among the 814 patients from 9 studies,^[13,14,16,18–21,24,28]^ the anastomotic leak rate in the stent group was higher than that in the emergency surgery group, with statistically significant differences (RR = 0.472, 95%CI: 0.286~0.777, *P =* .003). Please refer to Table 6 for details.

**Table 6:** Indicators related to surgical outcomes.

Effect size	Studys	numbers	Heterogeneity test	Analysis model	RR/SMD	95%CI	*P*
Surgical time	9	454	*P* = 0,	Random-effects model	−0.591	(−0.789, −0.392)	0
			*I*^2^ = 90.5%				
Cumulative blood loss	3	141	*P* = 0,	Random-effects model	−1.046	(−1.426, −0.666)	0
			*I*^2^ = 94.4%				
Transfusion rate	3	56	*P* = .948,	Fixed-effects model	0.624	(0.419, 0.930)	.021
			*I*^2^ = 0.0%				
Permanent ostomy rate	8	653	*P* = .230,	Random-effects model	0.625	(0.499, 0.782)	0
			*I*^2^ = 24.0%				
Overall ostomy rate	12	842	*P* = .096,	Fixed-effects model	0.520	(0.439, 0.614)	0
			*I*^2^ = 36.8%				
Number of lymPh nodes removed during surgery	5	412	*P* = .665,	Fixed-effects model	0.222	(0.021, 0.423)	.031
			*I*^2^ = 0.0%				
Primary anastomosis rate	9	814	*P* = 0,	Random-effects model	1.390	(1.030, 1.870)	.035
			*I*^2^ = 80.3%				

SMD = standardized mean differences.

##### 3.2.2.5. Other relevant indicators.

Eleven studies^[13–15,17,19–21,24,26–28]^ reported the total hospital stay of 763 patients, combined data shows that the total hospital stay of the stent group was less than that of the emergency surgery group, and the difference was statistically significant (SMD = −0.643, 95%CI: −1.159 to −0.128, *P =* .014). The summary results of 3 studies,^[14,20,28]^ including 119 patients, showed no statistically significant difference in total costs between the stent group and the emergency surgery group (SMD = 0.073, 95%CI: −0.299~0.445, *P =* .70); the summary results of 4 studies,^[13,20,25,29]^ including 141 patients, showed no statistically significant difference in ICU hospital stay time between the stent group and the emergency surgery group (SMD = −0.081, 95%CI: −0.744~0.582, *P =* .810), as shown in Table 7.

**Table 7:** Indicators related to other effect size.

Effect size	Studys	numbers	Heterogeneity test	Analysis model	RR/SMD	95%CI	*P*
Total cost	3	119	*P* = 0,	Random-effects model	0.073	(−0.299, 0.445)	.700
			*I*^2^ = 86.2%				
Total length of hospital stay	13	763	*P* = 0,	Random-effects model	−0.643	(−1.159, −0.128)	.014
			*I*^2^ = 90.4%				
The length of ICU stay	4	141	*P* = .051,	Random-effects model	−0.081	(−0.744, 0.582)	.810
			*I*^2^ = 66.3%				

SMD = standardized mean differences.

## 4. Discussion

Mechanical obstruction caused by CRC accounts for 55% of all colorectal obstructions, and 15%~29% of patients with CRC have intestinal obstruction as the initial symptom.^[31]^ Relief of obstruction and tumor clearance are still the most important aspects of treatment for obstructive CRC. Due to the presence of the ileocecal valve, colorectal obstruction becomes a closed-loop obstruction, which can cause a series of pathological and physiological changes, including electrolyte and acid-base balance disorders, intestinal ischemic necrosis, and impairment of intestinal mucosal barrier function leading to bacterial translocation.^[32,33]^ Emergency surgery in critically ill patients has a higher rate of medical history and complications. The placement of a self-expanding metal stent as a preoperative bridge can relieve the symptoms of acute intestinal obstruction and promote intestinal function recovery, allowing surgeons more time for comprehensive and detailed preoperative evaluation to improve the tolerability and prognosis of tumor resection surgery.

However, our meta-analysis found that self-expanding metal stents (SEMS) are not beneficial for the long-term oncological outcomes of curable diseases. This may be due to the adverse histological characteristics of tumors caused by stent placement, such as peripheral nervous invasion and lymph node invasion, which increase the risk of tumor spread.^[24]^ Our study summary data showed that the technical success rate of SEMS was 92.4%, and the clinical success rate was 80.9%. Previous studies have found that SEMS implantation, particularly when stent-related perforation occurs, can release cancer cells into the bloodstream, leading to the dissemination of tumor cells.^[34–36]^ Therefore, during stent placement, attention should be paid to minimizing complications such as stent perforation by performing fine and precise procedures, instructing patients on reasonable diets after surgery, and maximizing the bridging effect of the stent. Some studies have shown that stent compression of tumor tissue can lead to extracellular tumor cell sedimentation, and pressure can induce the production of hyaluronic acid, an adhesive molecule that promotes the progression of malignant tumors.^[37]^ Therefore, in order to reduce the risk of tumor spread caused by mechanical pressure and tissue erosion from the stent itself, the placement time of the stent should be minimized on the premise of improving intestinal symptoms. However, some studies have shown that the insertion of stents can lead to the spread of cancer cells into the blood, but these shed cancer cells may not be tumor stem cells and have a low malignant potential. Moreover, these cancer cells can be quickly recognized and killed by the immune system. Daily shedding of circulating tumor cells can number in the tens of thousands, but less than 0.01% of these cells survive in the end.^[34]^ Currently, guidelines recommend that these patients should undergo neoadjuvant chemotherapy before surgery, which not only increases the chance of negative tumor margins but also treats potential lymph node and distant micrometastasis to achieve downstaging, effectively eliminating these shed tumor cells.^[38,39]^ The latest research indicates that preoperative neoadjuvant chemotherapy combined with SEMS is a safe, effective, and well-tolerated strategy, characterized by a significant laparoscopic resection rate and a low stoma rate. However, the long-term outcome of this strategy remains uncertain.^[40]^ Additional research and extended follow-up are needed to gain a more comprehensive understanding of its long-term oncological outcomes.For distant metastases, there was no statistically significant difference in the incidence rate of liver and lung metastasis between the SEMS group and the emergency resection (ER) group. This may be because most patients with obstructive CRC have a late tumor stage, with local advanced infiltration or distant hepatic or pulmonary metastasis before treatment.

Previous meta-analyses have shown that the long-term results of 3-year and 5-year overall survival rate and disease-free survival rate were similar between SEMS and ER groups, but SEMS intervention had a better short-term therapeutic effect for surgery.^[41–43]^ However, the difference in 30-day mortality between the 2 groups is still controversial in various literatures. Spannenburg et al‘s meta-analysis found that the 30-day mortality rate in the SEMS group was lower than that in the ER group,^[44]^ but their study included a large number of non-randomized controlled trials, which may affect the authenticity of the results. Our study included all high-quality RCT studies, and found that there was no significant difference in 30-day mortality between the SEMS group and the BTS group, whether the purpose of treatment was palliative or curative.

Our study also found that the overall incidence of complications was lower in the SEMS group than the ER group, which is consistent with previous research results.^[11,45,46]^ By implanting a stent to relieve obstruction, not only can the overall stoma rate and permanent stoma rate be reduced, but also 1-stage laparoscopic intestinal anastomosis can be performed to achieve minimally invasive surgery, thereby improving the quality of life of patients. For incurable patients, palliative treatment can be performed by endoscopic stent placement to relieve acute intestinal obstruction; for curable diseases, SEMS implantation can shorten the surgical operation time after the recovery of intestinal function and thus reduce the incidence of surgical site infections. The decrease in the incidence of surgical site infections may also be related to the recovery of intestinal bacterial translocation after SEMS placement. In addition, our study also found that the cumulative blood loss and transfusion rate were lower in the SEMS group, which may be due to the reduced intraoperative blood loss leading to a reduction in the demand for blood transfusions. Patients can therefore recover better from surgery, thereby reducing the total hospital stay and ICU stay. In addition, cumulative blood loss as an important factor in the incidence of perioperative complications may be related to the low incidence of perioperative complications in this study.

It is worth discussing that the total number of lymph node dissections in the SEMS group was higher than that in the ER group, possibly due to the more thorough intestinal preparation and higher proportion of endoscopic surgery in the SEMS group, which provided a clearer view. Theoretically, the risk of overall tumor recurrence would decrease with an increase in the number of lymph nodes removed, but our research results were actually the opposite. This may be because the number of lymph nodes removed is not necessarily related to the number of positive lymph nodes.

This study must consider several limitations. Firstly, because the included patients only came from RCT data, there is a strong inherent selection bias. Secondly, due to differences in sample size, SEMS types, surgical methods and levels, tumor pathology types, research designs and follow-up times, there is heterogeneity between studies. Finally, but not least, because some continuous variables’ raw data is presented in the form of medians and quartiles, data errors are inevitable in the process of converting them into means and standard deviations.

In conclusion, this study has found that endoscopic stent implantation and emergency surgery have their own advantages and disadvantages. As a bridge to surgery, SEMS is superior to ER in short-term surgical outcomes for MLCO treatment. However, in terms of the overall tumor recurrence rate, SEMS may lead to poor long-term oncological outcomes. Nonetheless, as a preoperative bridge, a colon stent allows emergency surgery to be converted to selective surgery, allows for accurate preoperative staging and proper adjuvant therapy. The combination of neoadjuvant chemotherapy, which reduces tumor staging and kills tumor cells disseminated by stent implantation, may provide a new approach for the treatment of obstructive CRC.

## Author contributions

**Conceptualization:** Cui Zeng, Xiangwu Ding.

**Formal analysis:** Rumin Shang.

**Methodology:** Fei Lv.

**Project administration:** Xiangwu Ding.

**Software:** Rumin Shang.

**Supervision:** Cui Zeng, Xiangwu Ding.

**Validation:** Rumin Shang.

**Visualization:** Rong Fang, Xiaochang Tian.

**Writing – original draft:** Rumin Shang, Xiangming Han.

**Writing – review & editing:** Rumin Shang, Xiangming Han.
